# 2494: Have interdisciplinary collaborations increased over the last 10 years at Johns Hopkins University? Results of a pilot study

**DOI:** 10.1017/cts.2017.181

**Published:** 2018-05-10

**Authors:** Christine M. Weston, Mia S. Terkowitz, Daniel E. Ford

## Abstract

OBJECTIVES/SPECIFIC AIMS: The purpose of this study is to determine if the prevalence of interdisciplinary collaborations has increased over the past 10 years at 1 CTSA-funded institution. METHODS/STUDY POPULATION: We used Scopus to identify all articles published by authors affiliated with any of the Johns Hopkins Institutions for the years 2005, 2010, and 2015. We limited the search by the Scopus Field Codes “Subject Area” to biomedical science only, “Document Type” to articles only, and “Source Type” to journals only. We further eliminated all articles with 1 author or more than 10 authors. This resulted in 2800 articles for 2005, 3987 for 2010, and 4569 for 2015. After exporting the articles, we randomly selected 25 from each of the 3 time periods. Using the World Public Library Outline of Academic Disciplines as a guide, every author was assigned 1 of the following disciplines: Social Science (eg, Psychology), Basic Science (eg, Biology, Chemistry), Agriculture, Computer Science, Engineering, Medicine, Public Health, Nursing, or an Interdisciplinary field (eg, Genetic Medicine) based on their department and school affiliation. Articles with authors who belonged to 1 discipline only were considered single-discipline articles, and articles with authors in a least 2 different disciplines were considered “interdisciplinary.” RESULTS/ANTICIPATED RESULTS: Based on the results of an initial pilot study, in 2005, 24% of articles were interdisciplinary, in 2010, 20% of articles were interdisciplinary, and in 2015, 60% of articles were interdisciplinary. The large gap between the first 2 time periods (2005 and 2010) and the most recent (2015), suggests a possible pattern of increasing growth of interdisciplinary collaborations over time. Expanding this analysis to a much larger sample size will provide additional important evidence. DISCUSSION/SIGNIFICANCE OF IMPACT: Increasing emphasis is being placed on evaluating the effectiveness of the CTSA consortium in achieving its goals and on developing methods to gauge its success. Systematic methods that are easy to replicate across hubs are needed to better understand and track the evolution of scientific collaborations over time. This study outlines a process for determining whether one of the major desirable outcomes of the CTSA, notably the growth of interdisciplinary collaborations, can be determined through the analysis of authorship patterns. Further research is needed to confirm the generalizability of these results across other CTSA hubs.

